# Crossing the Cleft: Communication Challenges Between Neuroscience and Artificial Intelligence

**DOI:** 10.3389/fncom.2020.00039

**Published:** 2020-05-06

**Authors:** Frances S. Chance, James B. Aimone, Srideep S. Musuvathy, Michael R. Smith, Craig M. Vineyard, Felix Wang

**Affiliations:** ^1^Department of Cognitive and Emerging Computing, Sandia National Laboratories, Albuquerque, NM, United States; ^2^All-Source Analytics Department, Sandia National Laboratories, Albuquerque, NM, United States

**Keywords:** artificial intelligence, neural-inspired algorithms, neuromorphic, deep learning, artificial neural network

## Abstract

Historically, neuroscience principles have heavily influenced artificial intelligence (AI), for example the influence of the perceptron model, essentially a simple model of a biological neuron, on artificial neural networks. More recently, notable recent AI advances, for example the growing popularity of reinforcement learning, often appear more aligned with cognitive neuroscience or psychology, focusing on function at a relatively abstract level. At the same time, neuroscience stands poised to enter a new era of large-scale high-resolution data and appears more focused on underlying neural mechanisms or architectures that can, at times, seem rather removed from functional descriptions. While this might seem to foretell a new generation of AI approaches arising from a deeper exploration of neuroscience specifically for AI, the most direct path for achieving this is unclear. Here we discuss cultural differences between the two fields, including divergent priorities that should be considered when leveraging modern-day neuroscience for AI. For example, the two fields feed two very different applications that at times require potentially conflicting perspectives. We highlight small but significant cultural shifts that we feel would greatly facilitate increased synergy between the two fields.

## Introduction

Neural-inspired artificial intelligence (AI) is based upon the fundamental assumption that brain circuits have been optimized by evolution. While biological brains face different evolutionary constraints compared to modern-day computers, it stands to reason that further exploration of the brain's underlying mechanisms and using these mechanisms to inform emerging approaches to AI will capture aspects of cognition that are currently challenging for AI (see (Hassabis et al., 2017), (Aimone, 2019) for in depth discussions). Correspondingly, notable advances in artificial intelligence (AI), for example reinforcement learning [e.g., as used by AlphaZero, see (Silver et al., 2018)], the Transformer network (Vaswani et al., 2017), and deep convolutional networks (Krizhevsky et al., 2012), are based upon descriptions or theories of brain function. Currently, the direct path for incorporating modern-day neuroscience (which is increasingly designed for more detailed descriptions of brain circuits and mechanisms) into AI approaches is unclear, although the numbers of efforts focused on this challenge are growing. This article describes differences between the two fields that, if addressed, could significantly expand the path from neuroscience to AI to ensure the continued growth of neural-inspired AI.

How AI can best leverage modern-day neuroscience, and correspondingly, how modern-day neuroscience can best inform the field of AI remain open questions and active areas of discussion. One confounding factor is that the brain can be understood at multiple levels, all of which have impacted AI [for recent reviews see (Yamins et al., 2014; Sinz et al., 2019)]. At the phenomenological level (also referred to here as function level), efforts to include additional brain-inspired elements include attention (Mnih et al., 2014), episodic memory (Blundell et al., 2016), continual learning (Kirkpatrick et al., 2017), imagination (Thomee et al., 2007), and transfer learning (Pan and Yang, 2010). At a more mechanistic level, efforts remain centered on applying relatively standard training techniques to hand-crafted architectures incorporating novel neural-inspired elements [for example, see the incorporation of recurrence for visual processing in (George et al., 2017, 2018; Nayebi et al., 2018; Kar et al., 2019; Kubilius et al., 2019)]. Examples of efforts to include biophysical detail at the single-neuron or synapse level include spiking neural networks (Tavanaei et al., 2019), neurogenesis (Draelos et al., 2017), spine stabilization (Kirkpatrick et al., 2017), and context-dependent activation or gating of neurons (Masse et al., 2018; Rikhye et al., 2018). While there is certainly interest in incorporating additional neural-inspiration at multiple levels, approaches for doing so do not appear to be growing at the same pace as the wealth of neurobiological data being produced by the broader neuroscience community.

Identifying the appropriate “depth of understanding,” or level of abstraction, for describing how neural circuits implement cognition (or any other task) in a manner that facilitates incorporation into an AI model is one of the greatest challenges facing neural-inspired AI. In our opinion, a significant but subtle challenge arises from differing perspectives between the two fields, largely driven by the end-goal applications that drive each field. Neuroscience has been pulled by funding priorities toward a focus on identifying loci of dysfunction (i.e., in disease or disorders) for potential therapeutic targets. This translates to a culture that emphasizes defining and describing specific system components. AI applications, on the other hand, require demonstrated improvements on performance on a specific task. For neural-inspired AI, there is often a focus on problems for which human performance still exceeds that of computers (see “Challenges of bringing neuroscience to artificial intelligence”). For such problems, AI culture is primarily focused on understanding how a system produces a solution at an algorithmic-level, rather than understanding the underlying mechanisms or the biological neural architectures. In contrast to neuroscience, AI research experiences almost no pull along the form axis.

The view of these cultural differences is further complicated by a seeming abundance of riches—the fact that there are multiple levels across which the two fields may interact. Using visual processing as an example to highlight the cultural differences and differing foci between the two fields, we can describe “levels” of research using three fundamental questions to describe the impact of a particular research effort: (1) “What is it?,” or form, is defined as understanding the specifics of the components that comprise the neural circuit or neural network. (2) “How does it work?,” or mechanism, is defined as understanding how components of a network work together. (3) “What does it do?,” or function, is developing the “higher-level” description or abstraction of function. These three levels may be mapped to Marr's three levels for understanding an information processing system, implementation, algorithm, and computation, respectively (Marr, 1982); however here we have cast the levels as questions to highlight the viewpoint of a researcher interested in incorporating new information into models (whether for neuroscience or AI).

**Figure 1:**
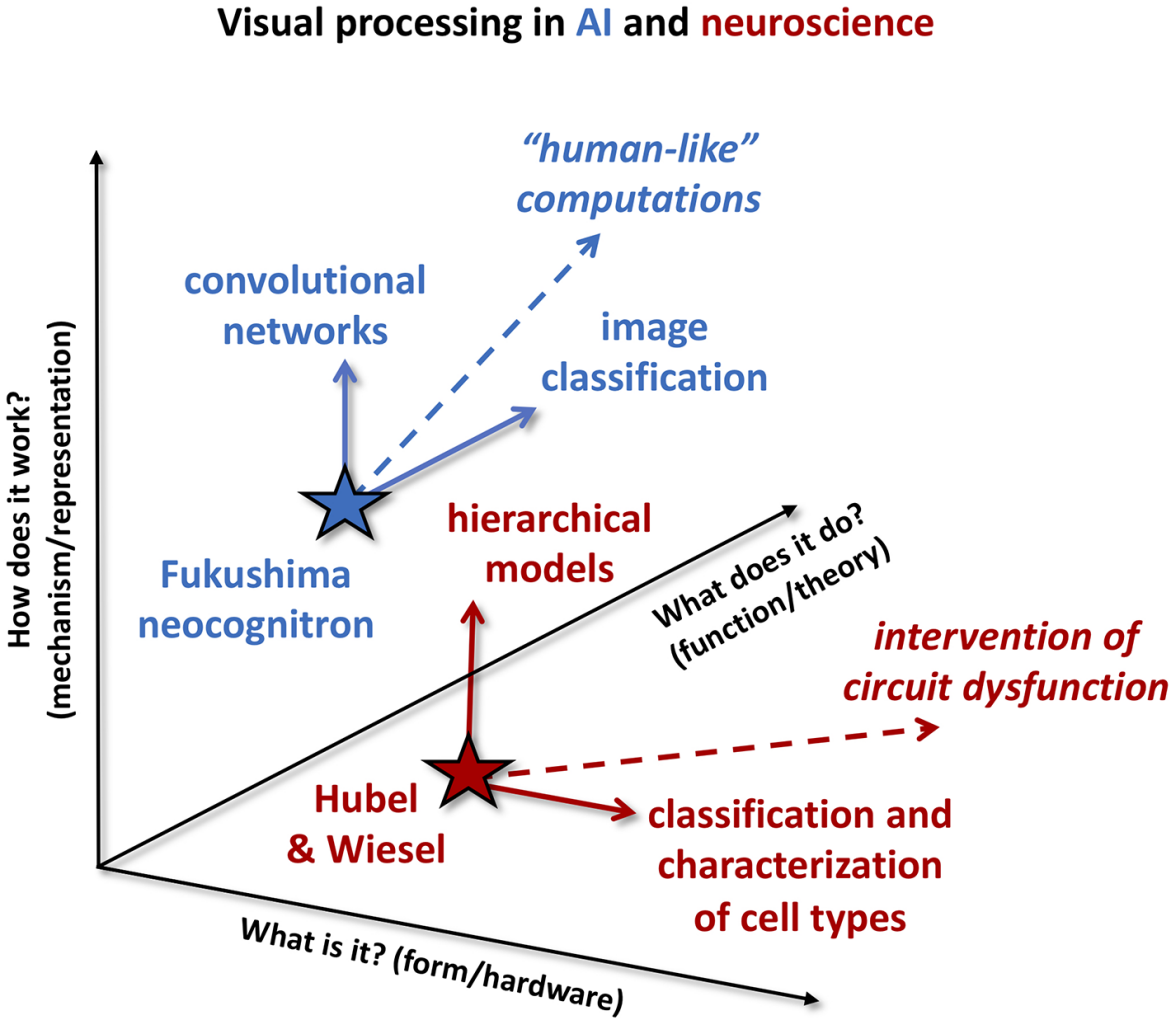
Cultural differences between AI and neuroscience. Example studies from visual processing in AI (blue) and neuroscience (red) are projected onto three different “axes” of impact: answering the question of “what is it?” (form or hardware), answering the question of “how does it work?” (mechanism or representation), and answering the question of “what does it do?” (function or theory). Neuroscience results tend to be communicated answering the “what is it?” or “how does it work” questions. As an example, Hubel and Wiesel's work (red star) characterizing simple and complex cells feeds continuing efforts along the form/hardware axis (horizontal solid red arrow) to further classify characterization of cell types in visual cortex. At the same time, Hubel and Wiesel's hierarchical model of visual processing has had significant impact along the mechanism/representation axis (vertical solid red arrow). Neuroscience experiences a strong application pull along the “what is it” axis, for example to identify therapeutic targets of circuit dysfunction (dashed red arrow). AI research tends to focus on “what does it do?” and “how does it work?” Here, development of Fukushima's neocognitron (blue star) into convolutional networks is illustrated as impact along the mechanism/representation axis (vertical solid blue arrow), while their application to image classification is impact along the function/theory axis (solid blue arrow). The dominant application pull on AI is to produce “human-cognition-like” computations (dashed blue arrow).

We illustrate these differences of our above example in Figure 1. As with Marr's levels, although the three axes are drawn orthogonally, we acknowledge that the axes are not completely independent, as any one experiment can impact multiple axes. For example, a combination of optogenetic labeling and large-scale calcium imaging can be used to characterize the responses of a specific subtype of neuron within a brain area. While describing the spatiotemporal features of this type of neuron's receptive field is form, inferring the underlying connectivity and interactions between cell types maps to the mechanism axis, and capturing a more abstract description of the functional role of this subtype within the larger population of recorded cells is aligned with the function axis.

While mechanism and function are both relevant for neuroscience, the drive for identifying therapeutic targets arising from a need for biomedical applications results in a strong pull predominantly along the form axis, as indicated by the red dashed arrow. This is not to say that all neuroscience work is only aligned along one axis. For example, the seminal work of Hubel and Wiesel (1962, indicated by the red star) characterizing receptive fields in visual cortex could be described as impacting two different axes. We would consider the ongoing efforts to further characterize responses and connectivity of various subtypes of neurons in V1 [for example see (Jiang et al., 2015)] as oriented along the form axis (what is it?), but the hierarchical model of visual processing (vertical red arrow) as oriented along the mechanism axis (how does it work?). The hierarchical model was fairly abstracted; it could be argued that this level of abstraction facilitated development of subsequent hierarchical models in AI, for example (Fukushima, 1980) neocognitron. Indeed, while many of the new tools becoming available today are specifically designed to address the form of large-scale neural circuits (see the next section, “Why neuroscience for artificial intelligence”), they will produce data that will drive new models at both the mechanism and function levels. Nevertheless, we would argue that orientation toward the “what is it?” question will continue to dominate, driven by the traditional applications of the field.

As with neuroscience, AI encompasses efforts aligned with all the axes described in Figure 1, but the drive for improving the performance on a specific task results in a significant pull along different axes than neuroscience. Algorithm development cannot proceed without some attention to mechanism (as well as implementation, see “Specialized hardware: agonist or antagonist?”), and often critical breakthroughs in performance arise from developing architectures. For example, the neocognitron (based upon Hubel and Wiesel's hierarchical model and indicated by the blue star), arguably inspired the architecture of convolutional networks [CNNs, (LeCun et al., 1998)], representing advancement along the mechanism axis. Nevertheless, answering the question of “what does it do?” (or perhaps put more colloquially, “what is it good for?”) is critical for applying a model to any application space. Training a convolutional network (e.g., for image classification, blue arrow) is application along the function axis. Similarly, implementing “human-like” computations (dashed blue arrow), like those thought to underlie cognition, while likely drawing from both mechanism and form, primarily will be oriented along the function axis.

Our intent is not to suggest that a bias along any one axis is more valuable than along another. However, the differences in Figure 1 are illustrative of why the two fields can sometimes be perceived as diverging, even as the fundamental research questions seem well-aligned. It is worth noting that neuroscience's general bias toward form and AI's general bias toward function may perpetuate a disconnect between the two fields, as each field will be predisposed to build upon advances framed along dominant biases of the field. For example, identification of a new type of interneuron (as would arise from further characterization of types of neurons in visual cortex) will not be readily incorporated into an existing machine learning approach or AI model without an accompanying *functional* description of the role of the interneuron within the biological neural network. Conversely, a generalized functional description of inhibition in an ANN may not be readily explored in a biological brain without some indication of form, or how that function might be implemented using known biological components (i.e., different interneuron subtypes).

In spite of cultural differences, there are indications that cross-pollination between the fields is thriving. Within the field of visual processing, it has been encouraging to see analogies drawn between the architecture of high-performing neural networks and visual cortex (e.g., George et al., 2017, 2018). Moreover, such comparisons have been extended to demonstrate that task-optimized deep convolutional networks appear to utilize representations similar to the single-unit responses of neural circuits contained within the ventral visual processing pathway (Khaligh-Razavi and Kriegeskorte, 2014; Yamins et al., 2014; Güçlü and van Gerven, 2015; Cadena et al., 2019). Several recent studies have proposed deep networks, trained to predict best stimuli for individual neurons, as validatable models of V1 (Walker et al., 2019) as well as higher-order areas of visual processing (Bashivan et al., 2019; Ponce et al., 2019). These works are examples of hybrid research, a product of both fields, that could facilitate development of a common language.

While a common language that spans both fields may be an ambitious goal, acknowledgment of differing priorities (or application drivers) may be the first step to subtle shifts in perspective that could do much to address the cultural differences between fields. For neuroscience, communicating new neuroscience knowledge on a function level will do much to ensure impact on AI. Similarly, a slight broadening of receptiveness of AI to differing levels of neuroscience would greatly facilitate adoption of new neuroscience knowledge. These shifts in focus are small but significant and would do much to increase the synergy between neuroscience and artificial intelligence.

## Why Neuroscience for Artificial Intelligence?

Neuroscience is in the midst of a technology development era that is producing new tools for exploring the brain's circuits with higher resolution and in greater detail than previously possible. First, recent advances in both electron microscopy (EM) imaging (e.g., Zheng et al., 2018), combined with novel reconstruction algorithms (e.g., Januszewski et al., 2018) are already resulting in new connectomes of unprecedented scale (e.g., Li et al., 2019), with even larger and higher-resolution volumes on the horizon. Potentially combined with other techniques such as the bar-coding of individual neuronal connections (Zador et al., 2012), neuroscience is now positioned such that a whole mammalian brain connectome is within reach. Second, and complementary to the large-scale connectomic datasets on the horizon, neuroscience also continues to advance large-scale calcium imaging (see (Girven and Sparta, 2017) and (Lecoq et al., 2019) for reviews) and multi-unit recording techniques, increasing the range of physical and temporal scales with which populations of neurons may be recorded (see (Stevenson and Kording, 2011) for a timeline). Third, a broader range of tools are now available for simultaneously identifying, recording and manipulating multiple populations from different cell-types [see (Huang and Zeng, 2013; Simpson and Looger, 2018)]. Detailed descriptions of interactions between different cell-types, including different temporal scales of plasticity, are essential for describing neuronal “motifs” that potentially constitute canonical computations in the brain (Douglas et al., 1989; Harris and Shepherd, 2015).

It would seem natural for the technological advances described above to drive a new and potentially revolutionary generation of neural-inspired ANNs. As increasingly accurate computational graphs of neurons become available, the question of how the brain is wired is no longer the limiting factor for developing novel and potentially revolutionary neural-inspired ANN architectures. Neuroscience now has the capability to record from the same populations of identifiable neurons for lengths of time that were previously unfeasible. Combined with advances in data analytics, neuroscience can now provide access to a range of neural temporal dynamics that were previously inaccessible. For example, the recent work by Trautmann et al. (2019) suggests that neural population dynamics can be extracted from silicon probe recordings without pre-requisite spike sorting, thus alleviating the data-processing bottleneck facing multi-unit recording techniques. These technological advances are particularly relevant from an algorithmic development point of view because the ability of a static graph (without corresponding knowledge of the temporal dynamics) to inform or constrain a computational neural model has historically remained unclear, as it is likely that temporal dynamics play a central role in biological neural processing. Nevertheless, the path from increased biological detail associated with large-scale recordings to a reduced form appropriate for incorporation into an artificial neural network is unclear. A neural model that reduces the temporal dynamics of these large-scale recordings to a more canonical form, even if at the expense of some biological detail, would do much to alleviate the disconnect (even if underutilizing the richness of the large-scale neurobiological data).

It is worth noting that Hubel and Wiesel's hierarchical model of simple and complex cells in visual cortex was a significant influence behind the development of the neocognitron, widely regarded as the predecessor to CNNs, even in the absence of anatomical validation. When considering the cultural differences raised in the previous section, one might even argue that the impact of Hubel and Wiesel's work was facilitated by the lack of anatomical validation as the hierarchical model was made accessible by its simplicity. Today computational neuroscience, driven by the availability of new large-scale datasets, is increasingly focused toward high throughput methods for data (Gouwens et al., 2019) to provide meaningful constraints for primarily mechanistic models. These efforts are synergistic with experimental neuroscience, as model validation often identifies critical gaps in knowledge. However, a more functional angle, potentially continued in parallel to the more detailed neural modeling, would do much to facilitate impact on AI.

## Challenges of Bringing Neuroscience to Artificial Intelligence

While AI researchers are highly motivated to explore novel approaches (e.g., neural-inspired architectures), that interest can fade without a relatively quick demonstrated impact on accepted benchmarks. In spite of the foundational work of Fukushima (1980) and LeCun et al., 1998), it was not until AlexNet won the ImageNet Large-Scale Visual Recognition Challenge (Krizhevsky et al., 2012) that CNNs rose to the level of popularity that they enjoy today. While it can be argued that the rise of CNNs was driven as much because availability of GPUs and large-scale data sets made training them tenable for the first time, their success and continued popularity is a significant example of how a concept, drawn from neuroscience and framed within the correct context (in this case tractability of training the network combined with success on a benchmark) can drive significant advances. It was the clear demonstration of function (successful application of the architecture) that drove the current and relatively widespread use of convolutional networks today.

In the case of AI, function is often defined by application. Broadly speaking, computer tasks may be divided into two categories: those for which a computer is currently better suited, and those for which a human is currently better suited. The latter category of tasks is an obvious desired application space for neuroscience, and AI has traditionally focused on improving performance in these areas while continuing to leverage capabilities for which a computer is better suited (e.g., extracting patterns from large corpuses of data). One example of a such a task is learning from a single or a few examples (zero-, one-, or few-shot learning). State-of-the-art algorithms currently achieve modest success at these tasks (Snell et al., 2017), but still remain unable to meet or beat human performance. A second, potentially related, task is extrapolating information to new examples (semi-supervised learning). Humans are able to recognize examples of a class of stimuli, even if presented in very different environments, after exposure to only a few labeled examples of that class with several unlabeled examples of that class and other classes. Developing algorithms that are capable of self-labeling new examples of a class remains a challenge for computer science [although see (Arjovsky et al., 2019)], presenting a real limitation to data processing algorithms as the process of labeling data is relatively expensive (and therefore large labeled datasets are not always readily available). Although these tasks are seemingly trivial for human beings, computer algorithms struggle to match human performance.

Demonstrating the value of looking to neuroscience for novel solutions to these tasks is particularly challenging, as neuroscience does not currently understand how the brain performs these tasks on the levels toward which neuroscience is biased. It is reasonable to assume that AI will be most strongly impacted by efforts aligned with the function axis (see Figure 1). Models of human behavior and human memory exist in a functional form, but they are relatively disconnected from studies at the neural circuit level (Krakauer et al., 2017). On one hand, this application space presents an opportunity for neural-inspired AI, as neuroscience will likely utilize approaches spanning all of the previously mentioned levels (form, mechanism, and function) to answer these questions. On the other hand, opportunities for neuroscience to impact this axis in the near future are constrained by the fact that most neuroscience tools available today are designed for exploring the form and mechanism of neural circuits (as previously discussed).

One practice that facilitates re-framing neuroscience form or mechanism data into a functional description appropriate for impacting AI is considering the functional context of the neural circuit (or single neuron) within the brain when assessing potential impact on AI. The majority of successful developments in neural-inspired AI (included those reviewed in this article) follow this practice. At the same time, we would encourage a broader perspective when considering which areas of neuroscience to draw from. A continued or increased focus on drawing from human cognition runs the risk of maintaining the disconnect between AI and neuroscience as the needed conversion from mechanistic and form descriptions to more functional ones may be slow to mature. In addition, our observation (discussed more fully in the next section) is that there are many opportunities for neuroscience to impact AI that will be overlooked without a broadening of the perceived “impact space” for neuroscience within AI.

## Crossing the Cleft

Currently a theoretical “gap” exists between neuroscience and AI as researchers seek to establish the “right” level of abstraction for translation between the two fields. While, as previously mentioned, the incorporation of neuroscience into AI development is often viewed as, at best, a superficial treatment of the understanding of neural circuits that neuroscience has to offer, neuroscience could do much to broaden its impact on AI through relatively small efforts to describe new discoveries in a function-oriented manner (answering the question of “What does it do?” in Figure 1), in addition to the form- and mechanism-oriented manners that are more common in the general neuroscience community. It is also worth noting that in some cases translation to a functional description may require loss of fidelity to the underlying mechanisms and form. Indications are that a cultural shift within computational neuroscience to describe brain theory in a more “machine-learning-accessible” manner has already begun [see recent papers by (Marblestone et al., 2016; Richards et al., 2019)]. As already described, neuroscience has also begun to adopt machine learning approaches to further develop computational models of neural systems, as seen for the visual system (Khaligh-Razavi and Kriegeskorte, 2014; Yamins et al., 2014; Güçlü and van Gerven, 2015; Bashivan et al., 2019; Cadena et al., 2019; Ponce et al., 2019; Walker et al., 2019).

In addition to better aligning with the goals of AI, from our viewpoint the impact of neuroscience on AI can also be extended by taking a broader view when considering what neural systems are relevant for fostering the development of neural-inspired AI (in particular those with more mature functional models derived from mechanistic and form data). One example of such an area is the exploration of visual processing in non-mammalian (but still strongly visual) animals. Recent work has identified neurons in the dragonfly visual system that exhibit a form of predictive gain modulation, in which visual responses to predicted prey-position are selectively enhanced (Wiederman et al., 2017), even in the presence of a second potential target (Wiederman and O'Carroll, 2013). Phenomenologically, the selective gain modulation of visual responses in the dragonfly system has obvious parallels with selective visual attention observed in macaque visual cortex (McAdams and Maunsell, 1999; Treue and Martínez-Trujillo, 1999). While the underlying neural circuitry and specific mechanisms are still under investigation in both the non-human primate and dragonfly systems, the relative simplicity of the dragonfly system has facilitated development of function-level models of the dragonfly mechanism [for example (Wiederman et al., 2008)] and subsequently development of dragonfly-inspired target tracking algorithms (Bagheri et al., 2015, 2017b) implemented on robotic platforms (Bagheri et al., 2017a).

A second example of the potential continued impact of neuroscience toward AI is the continued incorporation of elements of spatial coding as observed within the hippocampal formation into navigation algorithms. When place fields (O'Keefe and Dostrovsky, 1971) and head-direction cells (Taube et al., 1990a,b) were first characterized, they were accompanied by hypothetical functional descriptions of their roles in spatial coding. While abstract, these proposed functions facilitated their incorporation into robot navigation systems (Arleo and Gerstner, 2000) as well as SLAM (simultaneous localization and mapping) algorithms [e.g., RatSlam, (Milford et al., 2004)]. More than a decade later, the field continues to draw from neuroscience discoveries [(Zhou et al., 2018), (Kresier et al., 2018a,b)], including grid cells (Fyhn et al., 2004; Banino et al., 2018; Cueva and Wei, 2018), and 3-dimensional representations (Yu et al., 2019). While it remains to be seen whether the hippocampal spatial code is representative of a more general framework for cognition (Bellmund et al., 2018; Hawkins et al., 2019), advances in our understanding of the spatial navigation system of animals have clearly had continued impact on development of artificial brain-inspired navigation algorithms, with longer-term implications for autonomous or semi-autonomous navigation systems that will rely on some form of AI.

While these neural systems may be viewed as esoteric by some, the successes in these areas suggest that a common language (or at least a common perspective) is already being developed, even if restricted to certain applications in which neuroscience has had a demonstrated but limited impact. While it may be debatable whether modern neuroscience is poised to unravel the neural circuits underlying cognition, these examples illustrate that there are several avenues by which continued application of neuroscience to AI will (1) continue to grow communication between the two fields and (2) foster the development of neural-inspired AI application areas that could eventually form the foundation for more general neural-inspired AI.

## Specialized Hardware: Agonist or Antagonist?

One potentially complicating aspect to looking to a broader range of neural systems for impacting AI is the potential increase in computational cost. As discussed previously, the availability of modern high-performing computing platforms such as GPUs are a significant factor in the recent success of deep neural networks. While ideally neural-inspired algorithms should not be biased by the dominant computer architectures of the time, in practice the cost of applying an algorithm to particular application domains will be a consideration. For this reason, aspects of neuroscience that can be incorporated into a deep learning framework have an advantage for impacting AI in that they can be run on high-performing technology. While using a deep learning framework as a “best-practices” guideline may be beneficial in the short-term to foster communication between neuroscience and AI, an unfortunate side effect is that many neural systems with much to offer (for example the hippocampus) may contain architectures that are rather distinct from the hierarchical processing models that inspired deep neural networks.

For this reason, when looking more broadly within neuroscience to inspire AI, it will be useful to also look beyond current computing technologies to what technologies may be on the horizon. Recent years have seen an increased prioritization of neuromorphic hardware solutions for AI applications (Esser et al., 2015; Blouw et al., 2019; Severa et al., 2019) in addition to their long-proposed use for neuroscience modeling (Indiveri et al., 2011; Furber, 2012). Programmable neuromorphic hardware remains a somewhat immature technology compared to GPUs and CPUs, however there are now a number of technologies such as IBM's TrueNorth (Merolla et al., 2014) and Intel's Loihi chips (Davies et al., 2018) that have sufficient neurons to implement a variety of neural circuits, especially some of the more succinct circuits (e.g., the dragonfly system discussed above). The trade-off for this programmability is potential increased difficulty of implementation. Until these newer neuromorphic hardwares mature, it is likely that GPUs and other accelerators will continue to prove most effective for simple neural networks.

The effectiveness of GPUs at accelerating deep neural networks in some ways demonstrates that initial costs (for example increases in required computational power) may be acceptable when initially exploring new areas of neuroscience for impacting AI. Although neuromorphic technologies have demonstrated computational advantages, these advantages typically come with restrictions on the set of neural capabilities (e.g., leaky integrate-and-fire neurons) that may be effectively implemented. From our perspective, the neuromorphic hardware community is, in many respects, still searching for clear evidence of what aspects of the brain should be incorporated in hardware. Should potential computational advantages be demonstrated, there will be considerable interest in pursuing aspects of neural realism that can fully realize these advantages.

## Summary

We have discussed certain cultural differences between neuroscience and AI that, from our viewpoint, hinder cross-pollination between the two fields. While such cross-pollination is, in itself, a challenging proposition, much of these differences are driven by diverging priorities and perspectives rather than technical obstacles. Neuroscience is primarily focused toward understanding form, the components of biological neural circuits, and mechanism, how neural circuits work. New neural data, driven by a stream of new tools for dissecting neural circuits, will be described from this perspective. AI, on the other hand, seeks to increase performance (with respect to an objective function), especially on tasks where human performance still exceeds that of computer algorithms. While it is natural to look to neuroscience to inform the next generation of AI algorithms, AI requires information in a more abstracted language than neuroscience typically produces. Incorporation of neural elements be biased toward functional descriptions of neural circuits and the brain.

There are indications that there is already a cultural shift within neuroscience to communicate results on a more function-oriented level (although the fields have yet to arrive at an agreed-upon “common language”). Our view is that this slight shift in perspective will do much to facilitate translation of new neuroscience knowledge to AI algorithms. We also suggest that neuroscience impacts on AI could be enhanced by broadening the current perspective regarding what areas of neuroscience are relevant to AI. We have pointed to two example neural systems (spatial navigation in the hippocampus and visual processing in insects) that have been successful at maintaining an open pipeline to impacting ANN development and implementation in robotic systems and that, in our view, demonstrate the potential of “alternative” neural systems to inform AI. History (and hindsight) will eventually reveal the “right” source of inspiration and the correct language with which to communicate. Our current view is that there is tremendous potential for the two fields to work together synergistically, potential that can only be realized through broader exploration of a wide range of possibilities.

## Author Contributions

FC wrote the first draft of the manuscript. JA, MS, and FC wrote sections of the manuscript. All authors participated in discussions regarding the content of the manuscript, manuscript revision, and read and approved the submitted version.

## Conflict of Interest

The authors declare that the research was conducted in the absence of any commercial or financial relationships that could be construed as a potential conflict of interest.
